# The Risk of Retinal Vein Occlusion in Young Patients with Mental Disorders: A Nationwide Cohort Study

**DOI:** 10.3390/jcm12144874

**Published:** 2023-07-24

**Authors:** Ji-young Lee, Sheng-min Wang, Seung-hee Jeon, Min-hee Kim, Kyung-do Han, Young-jung Roh

**Affiliations:** 1 Department of Ophthalmology and Visual Science, Yeouido St. Mary’s Hospital, College of Medicine, The Catholic University of Korea, Seoul 07345, Republic of Korea; jiyounglee.md@daum.net (J.-y.L.); chriszz@naver.com (M.-h.K.); 2 Department of Psychiatry, Yeouido St. Mary’s Hospital, College of Medicine, The Catholic University of Korea, Seoul 07345, Republic of Korea; shengmin11@naver.com; 3 Department of Ophthalmology and Visual Science, Incheon St. Mary’s Hospital, College of Medicine, The Catholic University of Korea, Seoul 06591, Republic of Korea; jsh881107@hanmail.net; 4 Department of Statistics and Actuarial Science, Soongsil University, Seoul 06978, Republic of Korea

**Keywords:** retinal vein occlusion, mental disorders, depression, sleep disorder, anxiety disorder, nationwide cohort

## Abstract

We investigated the association between mental disorders and the incidence rate of retinal vein occlusion (RVO) in young Korean adults. This nationwide cohort study included subjects aged 20–40 years who underwent the Korean National Health Examination between 2009 and 2012. The diagnoses of RVO and mental disorders were based on the International Classification of Diseases Tenth Revision codes. Multivariate Cox proportional hazard regression models were used to evaluate the objective. In total, 6,891,399 subjects were included at baseline and 6,597,803 subjects (mean age, 30.86 ± 4.99) were finally analyzed for a mean follow-up duration of 7.36 ± 1.13 years, with the mental disorders group followed for 7.27 ± 1.15 years and the non-diagnosed group for 7.37 ± 1.12 years. Among a total of 10,145 subjects diagnosed with RVO, 1304 had been diagnosed with mental disorders, while 8841 had not. Cumulative incidence of RVO demonstrated a substantially higher incidence probability in subjects with mental disorders (log-rank *p* < 0.0001). Mental disorders were associated with an increased incidence rate of RVO (hazard ratio [HR]: 1.268; 95% confidence interval; [CI]: 1.196–1.344). In the subgroup analysis, subjects with depression, sleep disorder, and anxiety disorder exhibited an increased risk of incidence of RVO in all regression models (all *p* < 0.001). In conclusion, mental disorders and the incidence rate of RVO were significantly positively correlated in a Korean nationwide population-based cohort study. These findings suggest that mental disorders may also be associated with the pathophysiology of RVO in young adults.

## 1. Introduction

Retinal vein occlusion (RVO) is the second most common retinal vascular disease and an important cause of vision loss and blindness. In 2008, approximately 16.4 million people were suffering from RVO [1,2]. In patients with RVO, varying degrees of retinal vessel narrowing and irregularity lead to non-perfusion, which typically manifests as a sudden, painless loss of vision. The clinical manifestations of RVO span a broad spectrum, ranging from mild retinal venous dilation or tortuosity, and small retinal hemorrhages, to extensive areas of non-perfusion accompanied by severe macular edema and extensive retinal hemorrhages with cotton wool patches. Two types of RVO are defined, based on the site of vascular occlusion: central retinal vein occlusion (CRVO) and branch retinal vein occlusion (BRVO). Although some differences exist, CRVO and BRVO share many features and are often considered together [3].

RVO occurs more frequently in people over the age of 65 years, but it can also occur in young adults and can be a significant cause of vision loss [4]. In 2015, about 10% of individuals diagnosed with RVO were under the age of 40 [5]. The most relevant systemic abnormalities in young RVO patients are atherosclerotic diseases (i.e., hypertension, diabetes mellitus, and dyslipidemia) similar to those in older patients, but other systemic diseases should also be considered [6]. Some reports of young RVO patients have revealed an association with thrombophilia [7], antiphospholipid antibody syndrome [8], factor V Leiden mutation [9], hyperhomocysteinemia [10], and hyperviscosity syndrome [11], revealing that the risk factors for RVO in young patients are distributed over a broader field [12]. However, these reports were based on small retrospective studies or case reports. The exact pathogenesis of RVO in young adults has not been clearly identified but is thought to differ from that of RVO in older individuals [12,13].

Taking a new perspective, we focused on the impact of mental disorders on RVO because research has shown that changes in retinal function could indicate dysfunctions associated with mental disorders [14]. Furthermore, mental disorders are among the leading causes of the global health-related burden [15], making their importance increasingly evident. It is especially important to address mental health issues in young adult patients because this period is critical for developing and improving their mental health and wellbeing [16]. As a result, young adults are more susceptible to mental disorders, including life-threatening complications [17]. According to the Global Burden of Disease Study 2019, mental disorders resulted in 125 million disability-adjusted life-years globally, accounting for 4.9% of all diseases that contribute to disability-adjusted life years. Depressive disorders are the most common of the mental disorders, followed by anxiety disorders and schizophrenia. The former two account for the top-25 causes of disability worldwide, regardless of age, and for the top-15 causes in young adults [18,19]. Furthermore, the emergence of the COVID-19 pandemic has had severe implications on mental health and has exacerbated mental disorders, resulting in depressive symptoms, anxiety, post-traumatic stress, insomnia, denial, anger, and fear globally [20]. During the COVID-19 pandemic in 2020, the global prevalence of depressive and anxiety disorders increased, with younger age groups being more affected than older age groups [21]. Therefore, focusing on mental health impacts has become more essential.

It has been previously reported that younger patients with RVO have a higher prevalence of psychological stress [22]. The aim of this nationwide cohort study is to investigate the association between mental disorders and the incidence rate of RVO in young Korean adults using the National Health Insurance Service, in order to explore new possibilities related to RVO incidence in young patients.

## 2. Methods

### 2.1. Source of the Database

We performed a nationwide population-based retrospective cohort study using the Korean National Health Insurance (NHI) database maintained by the National Health Information Service (NHIS), a government-affiliated agency that administers the medical service system in Korea.

The NHIS encourages that all eligible Korean adults receive a regular healthcare checkup every two years, and the results are anonymously included in the NHIS database. The database covers almost the entire Republic of Korean population and includes general health examination records along with laboratory results, diagnostic data collected on International Classification of Diseases Tenth Revision (ICD-10) codes, medical expenses, use of inpatient and outpatient care, prescription records, and sociodemographic data [23].

This study was conducted in accordance with the tenets of the Declaration of Helsinki and approved by the institutional review board of the Catholic University of Korea (XC22ZIDI0021, Seoul, Republic of Korea). The informed consent was waived due to the nature of the study.

### 2.2. Study Population

Among all NHI beneficiaries, subjects aged over 20 to under 40 years who underwent the National Health Examination between January 2009 and December 2012 were included at baseline. Individuals with missing data and a prior history of RVO were excluded. A one-year lag period was incorporated from the date of the healthcare checkup to account for the duration of mental disorders.

### 2.3. Definition of RVO and Mental Disorders

Subjects with RVO were identified based on the assignment of ICD-10 codes H348, incorporating BRVO and CRVO. The study’s population comprised individuals who had received diagnosis of a mental disorder, identified by the presence of specific ICD-10 codes in their medical records within the five years prior to the health screening examination. Subjects with two or more mental disorders were not included in the study. The following ICD-10 codes were used to identify these disorders: codes F32–F33 for depression, F30–F31 for bipolar disorder, F20 for schizophrenia, G470 and F510 for sleep disorder, and F40–F41 for anxiety disorder.

### 2.4. Covariates

The baseline characteristics of the subjects, including demographic information and laboratory results, were recorded during regular medical checkups. Blood samples for the measurement of fasting plasma glucose, total cholesterol, high-density lipoprotein cholesterol, and triglyceride levels were obtained after overnight fasting before each examination. Blood pressure, waist circumference (WC), height, and body weight were measured. Body mass index (BMI) was calculated as kg/m^2^. Obesity was defined as a BMI exceeding 25 kg/m^2^, according to the Clinical Practice Guidelines for Overweight and Obesity compiled by the Korean Society for the Study of Obesity [24]. 

We also assessed the subjects’ lifestyle variables based on a self-reporting questionnaire. Current smokers were defined as those who had smoked 100 cigarettes in their lifetime. Heavy alcohol drinkers were defined as those who drank more than 30 g of alcohol per day. Intense exercise was defined as moderate-intensity activity more than five times per week, or vigorous activity at least three times per week. A low economic status was defined as the bottom 20% of medical aid and income.

Comorbidities were identified using medical history records, ICD-10 codes, and prescription codes. Diabetes mellitus (DM) was defined by ICD-10 codes E11–E14. with antidiabetic medications or fasting blood glucose levels (FBS) ≥ 126 mg/dL. Hypertension (HTN) was defined as ICD-10 codes I10–13 and I15, with antihypertensive medications, systolic blood pressure (SBP) ≥ 140 mmHg, or diastolic blood pressure (DBP) ≥ 90 mmHg. Dyslipidemia was defined as ICD-10 code E78, with antihyperlipidemic medications or a total blood cholesterol level of ≥240 mg/dL. Metabolic syndrome (MetS) was defined as the presence of ≥3 of the following: (1) WC ≥ 90 cm in men, ≥ 85 cm in women; (2) FBS ≥ 100 mg/dL or patients with DM; (3) SBP ≥ 130 mmHg or DBP ≥ 85 mmHg, or patients with HTN; (4) HDL ≤ 40 mg/dL in men, HDL ≤ 50 mg/dL in women, or patients with dyslipidemia; and (5) triglycerides ≥ 150 mg/dL or patients with dyslipidemia [25].

### 2.5. Statistical Analysis

Data are presented as the mean ± standard deviation for continuous variables and as numbers and percentages for categorical variables. An independent *t*-test was used for continuous variables, and a chi-squared test was used for categorical variables. Kaplan–Meier curves and log rank test for the cumulative incidence probabilities of RVO were generated. Cox proportional hazards regression models were used to evaluate the association between the incidence of RVO and the presence of mental disorders. Model 1 was unadjusted, and Model 2 was adjusted for age, sex, smoking status, alcohol intake, physical activity, and economic status. Model 3 was further adjusted for MetS. The incidence of RVO was calculated based on the number of events divided by 100,000 person-years. A subgroup analysis was conducted to determine the interaction *p* value, considering different baseline factor variables. Statistical analyses were performed using SAS version 9.4 (SAS Institute Inc., Cary, NC, USA). Statistical significance was set at *p* values < 0.05.

## 3. Results

**Figure 1:**
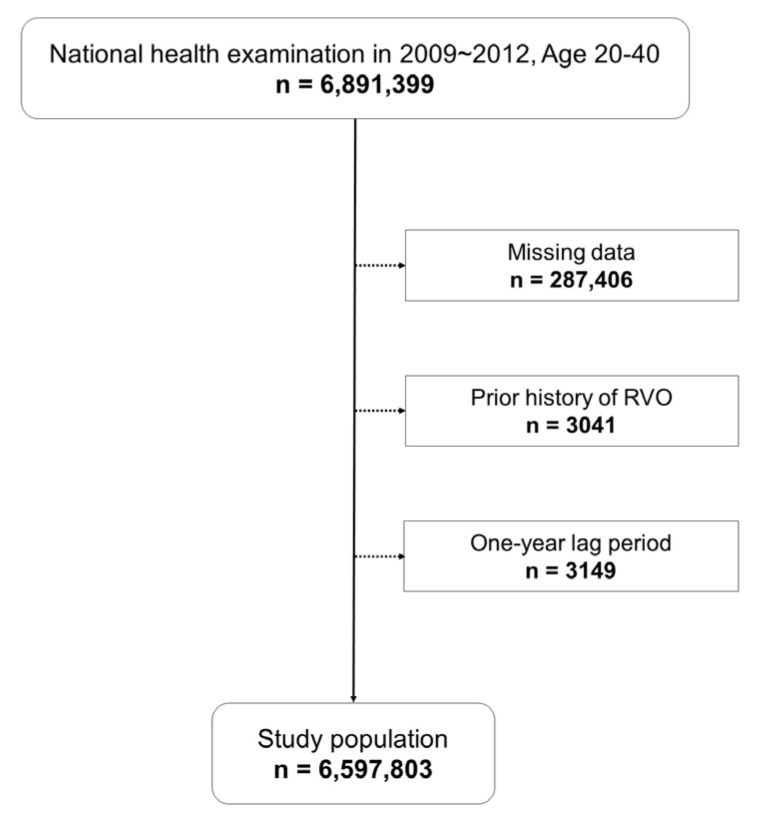
Flow diagram of study design.

In total, 6,891,399 subjects were included at baseline and those with missing data (*n* = 287,406), a prior history of RVO (*n* = 3041), and with one-year lag period (*n* = 3149) were excluded. Finally, 6,597,803 subjects (mean age, 30.86 ± 4.99 years; men, 59.6%) were analyzed in the study (Figure 1). All individuals were monitored until December 2018, with a mean follow-up duration of 7.36 ± 1.13 years, with the group with mental disorders being followed for 7.27 ± 1.15 years and the non-diagnosed group for 7.37 ± 1.12 years. 

Table 1 presents the baseline characteristics of the study population, according to the diagnosis of mental disorders. The mental disorders group included a significantly higher proportion of females and individuals over 30 years of age at the time of diagnosis. Subjects with mental disorders were more likely to have DM and dyslipidemia, and were less likely to have HTN and MetS than the non-diagnosed group. Various cardiometabolic parameters, such as BMI, WC, FBG, blood pressure, total cholesterol, and triglyceride levels, were significantly lower in subjects with mental disorders. The proportions of current smokers and heavy alcohol drinkers were higher in the non-diagnosed group, but the proportion of individuals with intense exercise and low income was higher in the mental disorders group.

**Figure 2:**
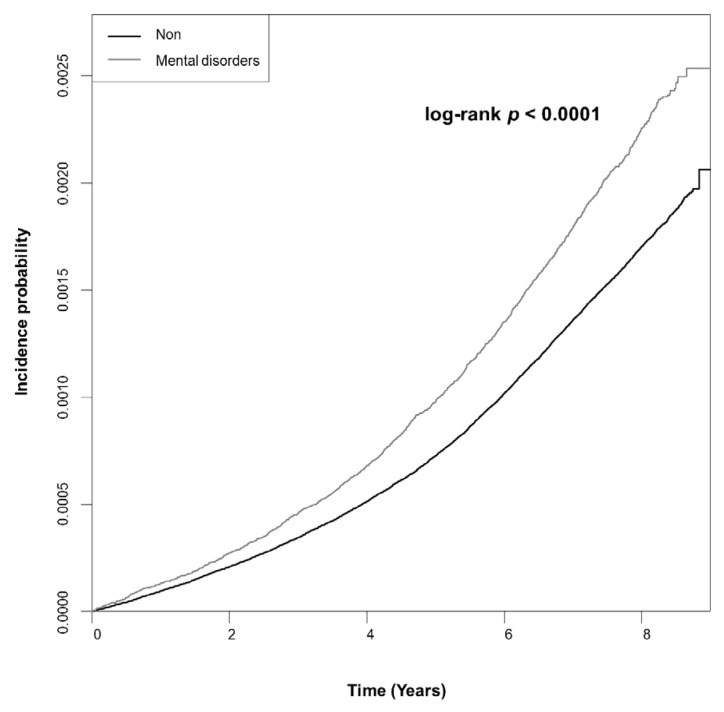
Kaplan–Meier curve for cumulative incidence of retinal vein occlusion in the mental disorders group and the non-diagnosed group.

**Figure 3:**
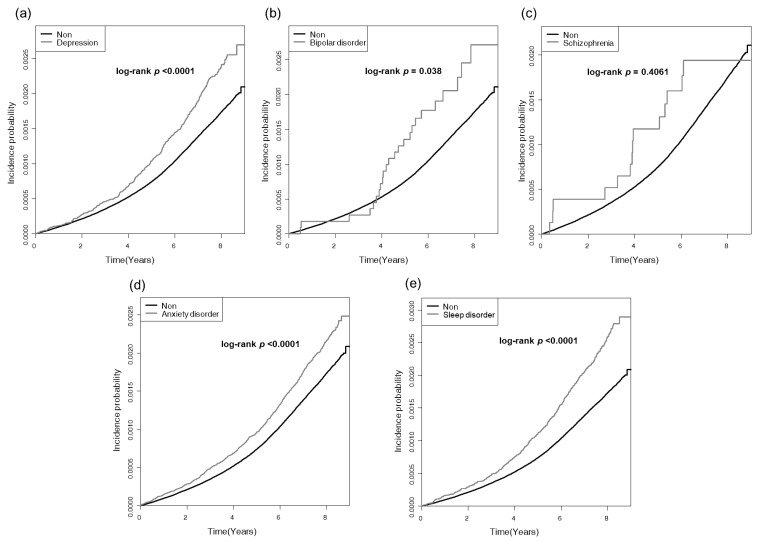
Kaplan–Meier curve for cumulative incidence of retinal vein occlusion (RVO) in the subgroup analysis. (**a**) Depression, (**b**) bipolar disorder, (**c**) schizophrenia, (**d**) anxiety disorder, (**e**) sleep disorder.

In total, 10,145 subjects were diagnosed with RVO, among whom 1304 had a prior diagnosis of mental disorders, while 8841 had not been diagnosed with mental disorders. Figure 2 shows the Kaplan–Meier curve for the cumulative incidence of RVO and demonstrated a substantially higher incidence probability in subjects with mental disorders (log-rank *p* < 0.0001). In the subgroup analysis, all subjects except those with schizophrenia showed a higher incidence probability relative to those in the non-diagnosed group (Figure 3).

Based on the multivariate Cox hazard regression models, Table 2 shows the association between mental disorders and risk of incidence of RVO. The adjusted HRs were 1.323 for Model 1 (95% CI: 1.249–1.402), 1.275 for Model 2 (95% CI: 1.202–1.352), and 1.268 for Model 3 (95% CI: 1.196–1.344) (all *p* < 0.0001), demonstrating a positive association between the risk of RVO and the number of individuals with mental disorders. In the subgroup analysis, subjects with depression, anxiety disorder, and sleep disorder exhibited an increased risk of incident RVO in all regression models: In depression, the adjusted HRs were 1.373 for Model 1 (95% CI: 1.239–1.522), 1.318 for Model 2 (95% CI: 1.189–1.462), and 1.308 for Model 3 (95% CI: 1.18–1.451). In anxiety disorder, the adjusted HRs were 1.258 for Model 1 (95% CI: 1.169–1.353), 1.212 for Model 2 (95% CI: 1.127–1.304), and 1.208 for Model 3 (95% CI: 1.122–1.299). In sleep disorder, the adjusted HRs were 1.493 for Model 1 (95% CI: 1.348–1.652), 1.418 for Model 2 (95% CI: 1.281–1.571), and 1.406 for Model 3 (95% CI: 1.269–1.557). (all *p* < 0.0001). Subjects with bipolar disorder showed a significantly increased HR only for Models 1 and 2. Subjects with schizophrenia did not show a significantly increased HR.

Table 3 shows the results of subgroup analyses for interaction *p* value based on various baseline variables. No significant interactions were observed with respect to any subgroup (all interactions, *p* > 0.05).

## 4. Discussion

In this nationwide population-based retrospective cohort study, we investigated the association between mental disorders and the incidence rate of RVO in young adults in the Korean population and found a significantly positive correlation. After adjusting for confounding variables, mental disorders were associated with a 26.8% increase in the risk of RVO incidence. In the subgroup analysis, depression, anxiety disorder, and sleep disorders significantly increased the risk of RVO incidence by 30.8%, 20.8%, and 40.6%, respectively, relative to the non-diagnosed group. 

Previously, Ha et al. [26] found that the presence of depression was significantly associated with an increased risk of RVO in nationwide cohort study. Our study has expanded the definition of mental disorders beyond depression, taking into account the global prevalence of mental disorders [18,19]. Additionally, we have specifically focused on young adults by narrowing the age range of the study subjects to provide a new perspective on the pathogenesis of RVO in younger patients, as it is thought to differ from the established risk factors seen in older individuals [12,13].

Based on its embryonic origin, the retina forms part of the central nervous system and exhibits similarities to the brain in terms of anatomy, functionality, and immunology [27]. Numerous mental disorders originate from aberrant brain neurotransmission [27]. So, recently, the retina emerged as a new way to explore cerebrovascular and neurodegenerative diseases [28] as well as brain abnormalities in mental disorders [29]. 

The retina and brain microvasculature have similar morphological and physiological properties, such as vascular networks, barrier circulations, and a regulatory circulation system [30]. Assessment of the retinal microvasculature is a useful method for visualizing the brain’s vascular network noninvasively [31]. Using the Parr–Hubbard formulas, retinal vascular calibers have been measured in several studies [32], and the retinal microvasculature has increasingly been shown to be an indicator of various mental disorders [33]. 

The pathophysiological mechanism between mental disorders and retinal microvasculature is not well established, but we hypothesize that psychological stress experienced by people with mental disorders [34] could have systemic impacts, such as endothelial dysfunction, inflammation, impaired glucose control, and hypoxia/ischemia, which are associated with changes in retinal vessel calibers [35]. Chronic psychological stress typically presents with hypothalamic–pituitary–adrenal (HPA) axis activation and upregulated catecholamine release through the autonomic nervous system, subsequently contributing to impaired endothelial dysfunction [36] and proinflammatory changes [37]. In a murine study, psychological stress induced the acute development of glucose intolerance and insulin resistance, with a rise in IL-8 related protein levels, which plays a key role in the recruitment of neutrophils [38]. In addition, chronic psychological stress impaired the recovery of mice from ischemic conditions and resulted in ischemia-induced neovascularization [39]. As well as microvascular changes, endothelial dysfunction might cause thrombosis because the compromised endothelial cells hardly prevent thrombus formation after injury originating from turbulent flow [3]. In addition, younger adults are more susceptible to psychological stress, depression, and anxiety symptoms [40]. As a result, the effects of these systemic issues may be more pronounced in individuals suffering from mental disorders at a young age.

Multiple studies provide evidence in support of this hypothesis. Psychological stress and negative emotions in children and adolescents are associated with larger retinal venules, which unfavorably reflect microvasculature abnormality [41]. Shalav et al. found that a wider retinal venular caliber was associated with worse neuropsychological functioning in middle age [31]. Caspi et al. found that individuals’ internalizing factors, indicated by depression, generalized anxiety disorder, and fears/phobias, were associated with wider retinal venules [42]. More specifically, depressive symptoms, through their impairment of mood regulation, are associated with cerebral microvascular damage which is related to retinal venular widening [43]. Poor sleep quality duration, especially inability to return to sleep [44], is associated with larger baseline central retinal venules.

In contrast to the findings of Appaji et al. [45], our study found no significant association between schizophrenia and the incidence of RVO. The vast majority of evidence suggests that schizophrenia is a result of neuronal maldevelopment, caused by disruptions to the developing brain induced by genetic or environmental factors as early as the late first or early second trimester and several markers of congenital anomalies have been found in schizophrenia [46,47]. Therefore, it could be surmised that individuals with schizophrenia would experience relatively lower levels of effects from psychological stress.

Meanwhile, subjects with bipolar disorder were associated with an increase in the risk of RVO incidence in some models. Appaji et al. also found that patients with bipolar disorder had significantly wider retinal venules and narrower arterioles than patients with schizophrenia [45]. Despite the fact that schizophrenia and bipolar disorder have considerable overlap in their pathophysiological processes and risk factors [48], other environmental and genetic factors might provoke differences in neurodevelopment between two diseases [49].

Our study had several limitations. First, there might be a selection bias for mental disorders in Korea because of the negative judgement on and stigmatization of mental disorders. In Korea, the use of mental health services is comparatively low because of negative attitudes toward the use of those services [50]. Indeed, many Asians consider mental illness to be a sign of weakness, and they tend to conceal their psychological problems [51]. Second, the incidence of RVO may have been underestimated because some asymptomatic patients with RVO would not seek medical care. Third, the diagnosis of RVO was based on ICD codes; therefore, the categorization of RVO (CRVO and BRVO) was limited, and there might have been some misclassification. However, BRVO and CRVO show some overlap in their risk factors, although they may have different phenotypes depending on the site of occlusion [52,53]. Fourth, the subjects with mental disorders in this study were individuals who had been diagnosed with mental disorders, validated by the presence of an ICD-10 code in their medical records within the five-year period preceding the health screening examination. Therefore, our study’s focus does not extend to investigating the association between the duration of mental disorders and the incidence of RVO. Finally, the study was conducted in the Republic of Korea and did not include other populations or healthcare systems. Despite these limitations, to the best of our knowledge, no previous study had investigated the association between various mental disorders and RVO in young adults, using large-scale nationwide cohort data. Furthermore, we comprehensively analyzed confounding variables and performed subgroup analyses that enabled adjustment for potential confounders.

## 5. Conclusions

In conclusion, this nationwide population-based cohort study demonstrated that mental disorders and the incidence rate of RVO were significantly positively correlated in young Korean adults. In the subgroup analysis, the incidence rate of RVO was significantly higher in patients with depression, anxiety disorders, and sleep disorders. These findings suggest that mental disorders may also be associated with the pathophysiology of RVO in young adult patients.

## Figures and Tables

**Table 1:** Baseline characteristics based on the diagnosis of mental disorders.

	Non-Diagnosed Group (*n* = 5,923,268)	Mental Disorders Group (*n* = 674,535)	*p* Value
Age (years)	30.8 ± 4.99	31.42 ± 4.99	<0.0001
20–29 (%)	2,524,133 (42.61)	253,333 (37.56)	<0.0001
30–39 (%)	3,399,135 (57.39)	421,202 (62.44)	<0.0001
Sex, male (%)	3,620,948 (61.13)	311,227 (46.14)	<0.0001
Current smoker (%)	2,112,635 (35.67)	192,330 (28.51)	<0.0001
Heavy alcohol drinker (%)	529,997 (8.95)	52,157 (7.73)	<0.0001
Intense exercise (%)	2,219,545 (37.47)	270,225 (40.06)	<0.0001
Low economic status (%)	917,240 (15.49)	122,873 (18.22)	<0.0001
DM	116,372 (1.96)	14,231 (2.11)	<0.0001
HTN	440,577 (7.44)	49,305 (7.31)	0.0001
Dyslipidemia	411,080 (6.94)	49,485 (7.34)	<0.0001
Metabolic syndrome	647,552 (10.93)	69,571 (10.31)	<0.0001
Obesity	1,593,096 (26.9)	161,717 (23.97)	<0.0001
BMI (kg/m^2^)	23.05 ± 3.61	22.69 ± 3.65	<0.0001
Waist circumference (cm)	77.69 ± 10.24	76.3 ± 10.58	<0.0001
FBS (mg/dL)	90.99 ± 16.9	90.7 ± 16.71	<0.0001
SBP (mmHg)	117.94 ± 13.2	116.16 ± 13.1	<0.0001
DBP (mmHg)	73.9 ± 9.47	72.92 ± 9.42	<0.0001
Total cholesterol (mg/dL)	184.92 ± 36.38	184.06 ± 36.1	<0.0001
HDL cholesterol (mg/dL)	57.53 ± 28.76	58.77 ± 27.85	<0.0001
Triglyceride (mg/dL)	120.25 ± 101.55	114.76 ± 97.45	<0.0001

DM, Diabetes Mellitus; HTN, Hypertension; BMI, Body Mass Index; FBS, Fasting Blood Sugar; SBP, Systolic Blood Pressure; DBP, Diastolic Blood Pressure; HDL, High Density Lipoprotein.

**Table 2:** Multivariate Cox hazard regression analysis of the association between RVO incidence and underlying mental disorders.

Mental Disorders		N	RVO	IR	HR (95% CI)		
					Model 1	Model 2	Model 3
Total	No	5,923,268	8841	20.256	1 (reference)	1 (reference)	1 (reference)
	Yes	674,535	1304	26.574	1.323 (1.249, 1.402)	1.275 (1.202, 1.352)	1.268 (1.196, 1.344)
					*p* < 0.0001	*p* < 0.0001	*p* < 0.0001
Depression	No	6,411,698	9768	20.691	1 (reference)	1 (reference)	1 (reference)
	Yes	186,105	377	28.037	1.373 (1.239, 1.522)	1.318 (1.189, 1.462)	1.308 (1.18, 1.451)
					*p* < 0.0001	*p* < 0.0001	*p* < 0.0001
Bipolar disorder	No	6,586,616	10,121	20.879	1 (reference)	1 (reference)	1 (reference)
	Yes	11,187	24	30.753	1.531 (1.027, 2.283)	1.496 (1.003, 2.23)	1.441 (0.965, 2.151)
					*p* = 0.0367	*p* = 0.0484	*p* = 0.0738
Schizophrenia	No	6,590,065	10,131	20.890	1 (reference)	1 (reference)	1 (reference)
	Yes	7738	14	25.534	1.249 (0.739, 2.109)	1.179 (0.698, 1.992)	1.132 (0.67, 1.913)
					*p* = 0.4063	*p* = 0.5375	*p* = 0.6419
Anxiety disorder	No	6,179,990	9362	20.573	1 (reference)	1 (reference)	1 (reference)
	Yes	417,813	783	25.704	1.258 (1.169, 1.353)	1.212 (1.127, 1.304)	1.208 (1.122, 1.299)
					*p* < 0.0001	*p* < 0.0001	*p* < 0.0001
Sleep disorder	No	6,422,836	9759	20.638	1 (reference)	1 (reference)	1 (reference)
	Yes	174,967	386	30.479	1.493 (1.348, 1.652)	1.418 (1.281, 1.571)	1.406 (1.269, 1.557)
					*p* < 0.0001	*p* < 0.0001	*p* < 0.0001
RVO, Retinal Vein Occlusion; HR, Hazard Ratio; CI, Confidential Interval							
Incidence Rates	100,000 person years						
Model 1	Non-Adjusted						
Model 2	Age, Sex, Current smoker, Heavy drinker, Intense exercise, Low economic status						
Model 3	Age, Sex, Current smoker, Heavy drinker, Intense exercise, Low economic status, Metabolic syndrome						

**Table 3:** Subgroup analysis on the association between mental disorders and RVO incidence.

Subgroup Factor		HR (95% CI)	*p* for Interaction
Age (years)	20–29	1.363 (1.201, 1.547)	0.1024
	30–39	1.244 (1.165, 1.329)	
Sex	Male	1.237 (1.145, 1.338)	0.4452
	Female	1.303 (1.192, 1.424)	
Smoking	No	1.256 (1.171, 1.348)	0.5717
	Current	1.289 (1.161, 1.432)	
Drinking alcohol	No	1.267 (1.191, 1.347)	0.8818
	Heavy	1.281 (1.055, 1.556)	
Intense exercise	No	1.28 (1.191, 1.376)	0.6836
	Yes	1.243 (1.124, 1.374)	
Low economic status	No	1.265 (1.187, 1.349)	0.7111
	Yes	1.272 (1.098, 1.474)	
DM	No	1.277 (1.201, 1.357)	0.406
	Yes	1.109 (0.907, 1.356)	
HTN	No	1.295 (1.215, 1.38)	0.0709
	Yes	1.095 (0.942, 1.273)	
Dyslipidemia	No	1.291 (1.213, 1.374)	0.0909
	Yes	1.071 (0.898, 1.276)	
Metabolic syndrome	No	1.318 (1.234, 1.406)	0.0555
	Yes	1.088 (0.952, 1.243)	
Obesity	No	1.293 (1.202, 1.39)	0.5504
	Yes	1.228 (1.113, 1.356)	

RVO, retinal vein occlusion; HR, Hazard Ratio; CI, Confidential Interval; DM, Diabetes Mellitus; HTN, Hypertension.

## Data Availability

Data available on request.
